# Computational identification of residues that modulate voltage sensitivity of voltage-gated potassium channels

**DOI:** 10.1186/1472-6807-5-16

**Published:** 2005-08-19

**Authors:** Bin Li, Warren J Gallin

**Affiliations:** 1 Department of Biological Sciences, University of Alberta, Edmonton, Canada T6G 2E9; 2 Department of Cell Biology, University of Alberta, Edmonton, Alberta, Canada; 3 Partners AIDS Research Center, Massachusetts General Hospital, Harvard Medical School, 149 13th Street 6th floor, Charlestown MA USA 02129

## Abstract

**Background:**

Studies of the structure-function relationship in proteins for which no 3D structure is available are often based on inspection of multiple sequence alignments. Many functionally important residues of proteins can be identified because they are conserved during evolution. However, residues that vary can also be critically important if their variation is responsible for diversity of protein function and improved phenotypes. If too few sequences are studied, the support for hypotheses on the role of a given residue will be weak, but analysis of large multiple alignments is too complex for simple inspection. When a large body of sequence and functional data are available for a protein family, mature data mining tools, such as machine learning, can be applied to extract information more easily, sensitively and reliably. We have undertaken such an analysis of voltage-gated potassium channels, a transmembrane protein family whose members play indispensable roles in electrically excitable cells.

**Results:**

We applied different learning algorithms, combined in various implementations, to obtain a model that predicts the half activation voltage of a voltage-gated potassium channel based on its amino acid sequence. The best result was obtained with a k-nearest neighbor classifier combined with a wrapper algorithm for feature selection, producing a mean absolute error of prediction of 7.0 mV. The predictor was validated by permutation test and evaluation of independent experimental data. Feature selection identified a number of residues that are predicted to be involved in the voltage sensitive conformation changes; these residues are good target candidates for mutagenesis analysis.

**Conclusion:**

Machine learning analysis can identify new testable hypotheses about the structure/function relationship in the voltage-gated potassium channel family. This approach should be applicable to any protein family if the number of training examples and the sequence diversity of the training set that are necessary for robust prediction are empirically validated. The predictor and datasets can be found at the VKCDB web site [1].

## Background

During the evolution of proteins there is interplay between selection acting to keep some residue identities constant, thus preserving protein function, and selection acting to accept new sequence variants with altered properties that confer improved survival. Thus, when studying the evolution of the structure-function relationship in a family of proteins, identification of invariant residues within the family identifies parts of the protein that are of central importance to its function. This idea is central to many comparative studies of protein structure/function relationships, and the concept has been extended to studies of pairs of residues whose identities co-vary in an apparently compensatory manner [2].

However, the converse idea, that varying residues are not centrally important to the protein's function, is not necessarily true. Although it is true that residues that do not have a major impact on protein function will show extensive variation over time, it is also true that residues that contribute to the quantitative variation in a protein's properties will also vary.

The problem that arises, then, is how to distinguish the residues whose variation in identity is responsible for functional variations in the protein from those residues whose variation in identity is relatively immaterial to function. These residues will not be detected by evaluating the extent of variation in a given residue or in pairs of residues. Rather, the residues will co-vary with the property of the protein that they affect. To solve this problem it is necessary to use techniques that can detect associations between combinations of residue identities at any position in the protein and the quantitative value of the parameter of interest. We here report an analysis to detect such an association between structure and function in voltage-gated potassium channels (VKCs) using machine learning techniques.

VKCs are membrane proteins that regulate the passage of potassium ions through membranes [3]. When the voltage difference across a membrane reaches a threshold, the probability that VKCs will open begins to become significant, allowing increased potassium ion diffusion through an ion-selective pore in the channel. This voltage-regulated potassium ion permeability is critical in cellular excitability. Mutations in VKC genes have been shown to be associated with cardiac arrhythmias [4], episodic ataxia [5], and other diseases [6,7]. Consequently, VKC proteins have been considered good targets for drug design directed at a number of diseases [8-10].

A functional VKC consists of four subunits, each containing six transmembrane regions, S1 through S6. S4 has been shown to function as the main voltage-sensing domain [3], acting by moving perpendicular to the plane of the membrane upon depolarization [11,12]. This movement causes a conformational change in the region of the pore to open the "gate" and allow potassium ions to pass through. There is currently heated dispute over which of several mechanisms that have been proposed to explain how the sensor movement changes the channel conformation is correct [13].

Elements of the molecular basis of VKC function have been elucidated through structural studies [11,14-18]. The structures of several potassium channels of different types have been determined crystallographically [11,15,17,19]. The structure of the ion selective pore is very similar in all of the models; these studies have clearly identified many aspects of the molecular dynamics of selective ion permeability. Although the structure of one voltage-gated potassium channel has been determined [11], the unusual mobility of the voltage sensor region and the necessity of using a bound antibody to stabilize the crystallized conformation raise serious questions of how similar that determined structure is to the functional conformation of the ion channel [20]. Extensive mutagenesis and biophysical studies of different voltage-gated ion channels have lead to several models of function that are quite different from each other and from the model proposed based on the KvAP crystal structure (KvAP structure ref). Thus, although the molecular basis of ion selectivity and permeability is well supported by the current structural knowledge, the molecular basis of voltage sensing, and in particular the molecular basis of the fine differences in voltage sensitivity between channels, is not well defined by structural studies.

In the absence of three-dimensional structures of various VKCs that unambiguously show different opening/closing stages, mutagenesis of individual residues of different VKCs has been the main method for inferring the structure-function relationship of VKCs. However, it is prohibitively time-consuming and costly to do mutagenesis of all residues individually and in combinations in different VKCs. Computational tools, usually multiple sequence alignment, have been used to identify conserved regions of VKCs and limit the priority in mutagenesis experiments to evolutionarily conserved residues [21-23]. Unfortunately, details of the complex structure-function relationship between individual residues and the electrophysiological properties, which are mostly continuous quantitative parameters [24], are too complicated to understand by simple inspection of aligned VKC sequences. With dozens of VKC sequences of a few hundred residues each and continuous electrophysiological variables, more mature data mining tools, such as machine learning, are necessary.

Machine learning generalizes an underlying data model for a phenomenon by "learning" from existing data from specific examples of that phenomenon, using various classification rules. It yields a mathematical or computational model that can best describe the existing data and predict classifications of new data [25]. Because of its ability to extract complex models from large datasets, machine learning has been successfully applied to many data-rich problems such as marketing reports, weather prediction, automatic genome annotation and microarray data analysis [26-29].

In this report we tested several learning algorithms, as implemented in the WEKA program package [30]. Most of these were categorical learners; they evaluate how features can be used to assign each sequence to a pre-defined discrete category. The OneR classifier identifies a set of rules based on the identity of the amino acid residue at only one of the various aligned amino acid positions that best classifies each sequence in the training set. The Decision Tree classifier identifies a minimal set of amino acid positions, and branching decisions based on residues at these positions, that correctly classify the training data. The Naïve Bayes classifier uses observed frequencies of residues at selected positions to apply Bayes' theorem to make probabilistic predictions of the category to which a sequence belongs. Kernel Density estimation estimates the probability distribution for use in the Bayesian analysis if a non-normal distribution is suspected. The K Nearest Neighbour (KNN) classifier was used for both categorical classification and for classification that treats V_50 _as a continuously varying quantitative characteristic; this method uses a distance measure to determine which elements of the training set are closest in attribute space to the example being evaluated and assigns the average of the k examples that are most similar to the test example as the predicted value.

Typically, a protein family comprises dozens of members with hundreds of residues in each member. Such datasets present a unique type of problem for machine learning. First, a typical training dataset for machine learning contains distinctively labelled "features" in every instance. With protein sequence datasets, all sequences must be aligned with each other to identify homologous residues (features). Second, dozens of sequences with hundreds of residues each create a dataset with very high dimensionality, which compromises learning performance. Finally, besides generating a classifier with high accuracy, it is pertinent to bench biologists to evaluate the biological importance of individual residues (features) that contribute to a good learning performance during training, so it is desirable to use learning methods that return the basis for their prediction.

In this report, we have mined available VKC sequence and electrophysiological data using machine learning and related feature selection techniques, and derived a model that predicts one of the central electrophysiological parameters, half activation voltage (V_50_) [24], of a given VKC, based on only its amino acid sequence. Our best result was obtained using a k-nearest neighbor classifier (k = 1) combined with feature selection using a wrapper algorithm [31], yielding a mean absolute error (MAE) between the predicted and published V_50 _values of 7.0 mV in a repeated ten-fold cross validation. The prediction by our final predictor was validated by permutation test and by comparison of predictions to independently obtained experimental results. The training process also provides a rational basis for identifying residues potentially critical to the activation of VKCs, and several identified key residues are located in regions that have been proposed to modulate VKC activation.

The methods that we have applied to the study of VKCs are general. With appropriate alignment, feature selection and model validation, this analytical approach can be used to generate biological hypotheses in other protein families and these hypotheses can be practically tested using site-directed mutagenesis.

## Results

### Learning without feature selection

**Figure 1:**
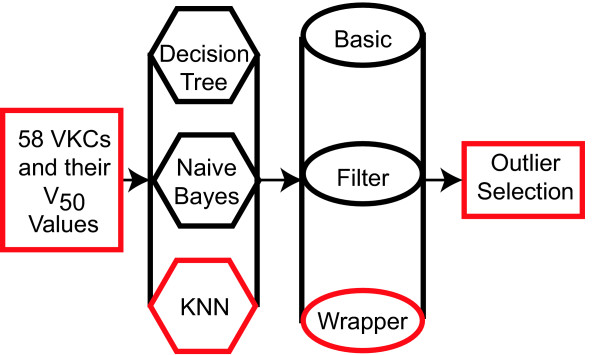
**Flow chart of procedures followed to develop the optimal V_50 _predictor**. The data set was subjected to several different learning algorithms, either alone or in combination with two types of feature selection. The KNN learning algorithm and the wrapper feature selection algorithm (highlighted in red) were found to yield the best results. These algorithms were then used to evaluate the effect of removing sequences as outliers to yield the data set used for construction of the final predictor. The individual processes that were used to construct the final predictor are highlighted in red.

**Figure 2:**
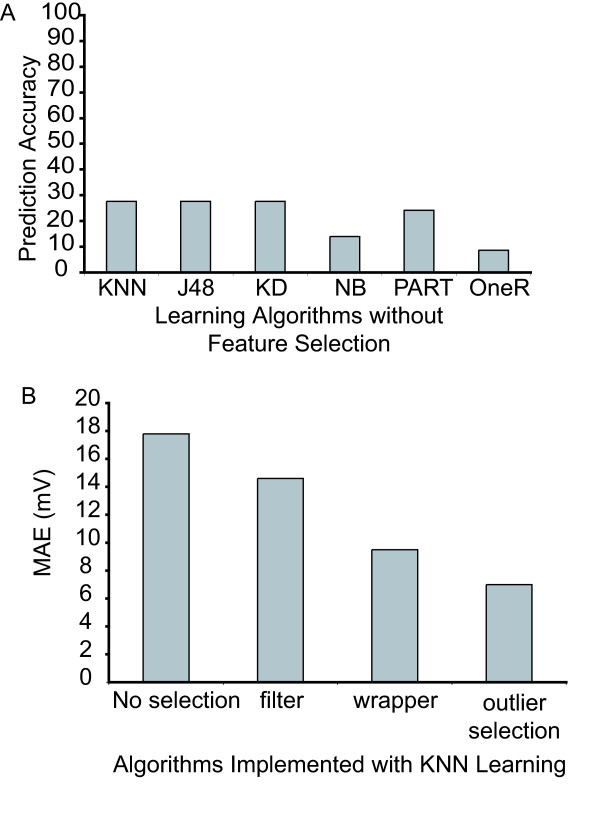
**Learning performances with different algorithms and different implementations**. All bars represent results of a repeated (ten times) ten fold cross validation. A: Categorical learning with different learning algorithms without feature selection. The V_50 _values were divided into seven classes based on their values. The learning was done without feature selection. B: Improvement of KNN prediction accuracies in different implementations. Results of KNN classification without feature selection, with the filter algorithm, with the wrapper algorithm, and with outlier selection in combination with the wrapper algorithm are shown. Both feature selection algorithms improved learning performance. The best learning accuracy was obtained using the KNN classifier combined with wrapper and removal of four outlier sequences. It yields a mean absolute errors of 7.0 mV with the new dataset (Dataset 2) of 54 VKC sequences.

A dataset consisting of 296 aligned positions from 58 VKC sequences (Dataset 1) was initially used to train different learning algorithms to predict the V_50 _value of a given VKC sequence. Figure 1 illustrates schematically the process that was used in developing the final predictor. V_50 _values were divided into seven nominal classes for categorical learning. The accuracy of the best categorical learning was below 30% (Figure 2A). The MAE of the best numerical prediction of V_50 _values with the KNN classifier (Figure 2B) was close to 18 mV. This analysis is equivalent to assigning a predicted V_50 _to a channel based on the V_50 _of the channel's nearest neighbor or neighbors in a distance-based phylogenetic tree. Evidently, these learning algorithms alone do not produce an accurate model for prediction if they are trained with such a high dimensional dataset of less than 60 instances. This is likely because a relatively small number of residues affect the V_50 _of a channel and the majority of residues affect other parameters, which do not co-vary with V_50_, of the channels.

### Learning with data filtering

To improve learning performance with this high dimensional dataset, we added feature selection before learning, using a filtering algorithm to decrease the dimensionality. All residues (features) were ranked based on their information gain scores [32,33]. Different numbers of top-ranked residues were then used for learning. The best learning performance was obtained using only the top five ranked features (residues), and the categorical accuracy improved to 36%. The MAE of the numerical prediction of V_50 _values with a KNN classifier was now reduced to 15 mV (Figure 2B). While dimension reduction by filtering to select residues with high information gain did appear to yield a better learning performance, the improvement is marginal.

### Learning with wrapper

We also applied a wrapper algorithm, a more learning performance-driven feature selection method than filtering [31]. From a large number of sets of residue (feature) combinations, wrapper selected the residue set that yielded the best learning performance. The prediction accuracies with all categorical learning algorithms improved, with the best classification of 60% accuracy using the KNN classifier. When the KNN classifier (k = 1) was combined with wrapper to predict a numerical V_50 _value based on a VKC sequence, the MAE of prediction improved to 9.5 mV from 17.8 mV (Figure 2B). The best prediction accuracy was obtained with six residues (features).

### Effect of scoring matrix choice and distance formula

We used an identity matrix (Formula 1.1) and transformed BLOSUM62 and PAM100 amino acid matrices (Formulas 1.0 and 1.2) for calculating distances in KNN classification. The best MAEs were the same for all three scoring matrices in repeated ten-fold cross validations. The best predictors obtained with each scoring matrix used 6 features (BLOSUM62), 8 features (PAM100) and 9 features (identity matrix). We performed the remainder of our analyses using the BLOSUM62-based matrix because it yielded the best accuracy with the fewest features. Five of the six features identified with the BLOSUM62-based matrix were identified by analyses with at least one of the other two scoring matrices (Table 1).

We also evaluated the result obtained by summing the individual character distances (Manhattan distance) to the result obtained using the Euclidian distance as described in Materials and Methods, using the transformed BLOSUM62 distance matrix. We obtained the same MAE with the same six selected features, indicating that the learning method is robust to the distance calculation method, at least with this data set.

### Learning combined with outlier selection

**Figure 3:**
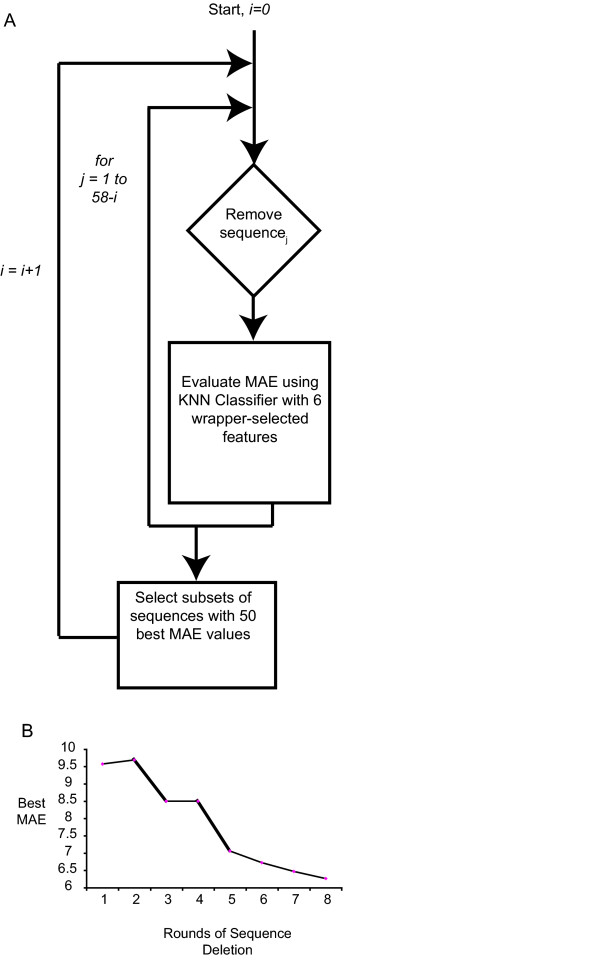
**Schematic of the process of outlier selection, and the variations in MAEs during outlier selection using KNN classifier**. A: Each sequence of Dataset 1 was individually deleted to select the resulting datasets that produce improved learning performances. The top 50 new subsets were kept at each round, and individual deletions were repeated. Due to computational complexity, the best feature set selected by wrapper as described in the paper was used in training. B: Variation of learning performance using KNN classifier during outlier selection. The mean absolute errors of prediction improved with selective removal of putative outlier instances. There was a significant improvement of learning accuracies at Round 2 and 4 (thick lines). After Round 4, the improvement of learning performances slowed down significantly.

Since the dataset has only 58 VKC sequences, a small number of outliers, or incorrect class labels, might have greatly affected the training process and thus led to poor learning performance. We evaluated the effect of deleting each sequence from the dataset, by training the KNN classifier with each of the 58 possible subsets of 57 sequences. The top 50 subsets with 57 VKC sequences that produced best learning performances using a repeated ten-fold cross validation were kept and the pruning procedure was then repeated with each of the 50 subsets as a starting point (the flow diagram for this process is shown in Figure 3A). The six-feature set that gives the best learning performances using Dataset 1 (MAE = 9.5 mV) was used during outlier selection. In spite of the plateau in Round 1 and 3, there were significant improvement of learning accuracies in Round 2 and Round 4. After four pruning rounds the improvement in accuracy significantly slowed down in the following rounds (Figure 3B). Thus, we believe that Round 1–4 represents informative gains in accuracy from deleting true outliers, whereas the improvement in later rounds is due to over fitting. This outlier selection improves the MAE from 9.5 to 7.0 mV

During the pruning process, four VKC sequences, VKC8 (Kv1.3 mouse), VKC98 (Kv1.4 dog), VKC149 (Kv2 squid), and VKC171 (Kv4.3 mouse) [34], were consistently selected as outliers from Rounds 1–4, although the order by which they were deleted varied. We therefore created a new dataset of 54 sequences (Dataset 2). The new dataset was used to construct the KNN final classifier, for which the best MAE improved to 7.0 mV (Figure 2B). We also re-ran the complete training protocol using the wrapper algorithm with Dataset 2, and exactly the same feature set was again selected, producing the best MAE of 7.0 mV.

We also evaluated two other measures of prediction quality, the R-squared value, which represents the percentage of variance in the training set that is explained by the predictor, and the correlation between the actual and predicted V_50 _values. For Dataset 1 the R-squared value for all characters is -0.18 and the correlation coefficient is 0.28; the R-squared value for the selected six features is 0.64 and the correlation coefficient is 0.79. For Dataset 2 the R-squared value for all characters is -0.09 and the correlation coefficient is 0.36; for the selected six features the R-squared value is 0.77 and the correlation coefficient is 0.89.

### Permutation tests

One hundred permuted datasets were generated by randomly shuffling the V_50 _values among the VKC sequences in the Dataset 2. With the same parameters and settings with which we obtained the predictor, we applied KNN classification combined with the wrapper algorithm for feature selection to each one of these permuted datasets, yielding 100 different predictors with different feature sets and performance values. These 100 replicates provide an estimate of the probability distribution of the MAEs for the null hypothesis that there is no functional relationship between the sequence and the V_50 _value. The MAEs with the permuted datasets range from 9.9 mV to 15.4 mV (mean = 13.4 mV, SD = 1.1 mV). The performance of the predictor with the original dataset (MAE = 7.0 mV) is significantly better (P < 10^-10^) than would be expected if there were no connection between the sequence and V_50 _value.

### Validation of predictor with independent experimental data

Thirteen wild type VKCs that were not part of the training data were evaluated with the final predictor. The V_50 _values of these "new" VKCs had been determined independently in electrophysiological experiments [34,35] (Salvador-Recala V, Gallin WJ, Abbruzzese J, Ruben PC, Spencer AN: A Kv4 channel cloned from the heart of the tunicate *Ciona intestinalis *and its modulation by a KChIP subunit. Manuscript submitted). The MAE of these predictions is 9.7 mV (See Additional file 2). Within this test set, two VKCs are from species that are evolutionarily distant from any of the other sequences in the training dataset, *Hirudo medicinalis *and *Ciona intestinalis *[36,37], and the prediction errors for these two VKC are both over 27 mV (Table 2). When the most distant sequence *(H. intestinalis*) is removed, the MAE of the remaining twelve VKCs is 8.3 mV, and if both sequences are removed MAE = 6.6 mV, below the MAE we estimated using a repeated ten-fold cross validation (7.0 mV).

We also evaluated the predictor by comparing the predicted V_50 _values of a number of VKC mutants with experimental data from an alanine mutagenesis scan of rat Kv2.1 by Li-Smerin *et al *[38]. The comparison is shown in Table 2. The MAE between our predictions and data obtained experimentally is 7.5 mV, which is reasonably close to our estimated MAE of 7.0 mV using cross validation. Note that in five of the cases the predicted values for V_50 _are the same; this is because none of the mutations to alanine makes a new set of informative sites that is closer to a new training sequence. In the two cases where one of the informative residues was mutated to a residue that is represented in one of the channels in the learning set (see Table 2), the prediction improves significantly (MAE = 1.35 mV, n = 2) (Table 2).

### Identification of informative features (residues)

**Figure 4:**
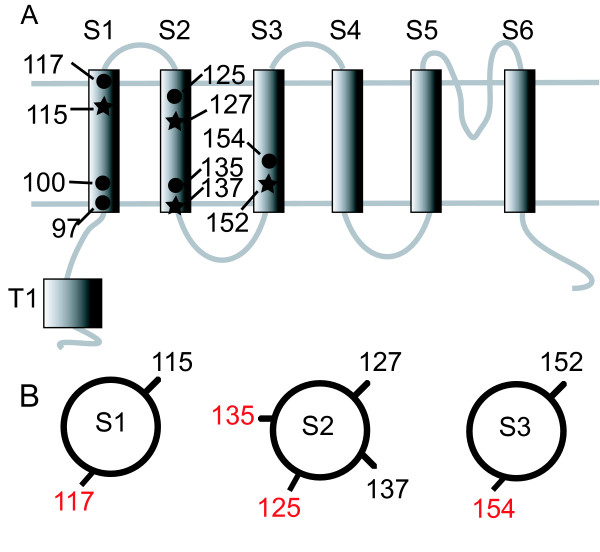
**Residues selected as informative features by wrapper and mapped onto VKC structures**. A. Schematic structure of the VKCs. Six transmembranehelices, labeled S1 through S6, traverse the plasma membrane. The N-terminus and C-terminus are both cytoplasmic. The loop connecting S5 and S6 folds into the channel to form the ion-selective pore. The S4 region, in which every third residue is positively charged, is the central voltage-sensing element of the VKC. The six residues selected by the wrapper algorithm are approximately mapped onto the schematic diagram (black dots). Four highly conserved acidic residues are indicated by black stars. The numbers assigned to each marked residue are positions within the dataset alignment, not positions in any actual VKC protein sequence.B. Schematic end-on view of helices S1, S2 and S3 with the radial positions of conserved acidic residues (numbered in black) and four of the selected residues (numbered in red) indicated. Residue 117, 125, 135 and 154 are each exactly two residues away from a highly conserved negatively charged residue, 115, 127, 137 and 152 respectively.

The wrapper algorithm identifies a relatively small number of residues that are the primary determinants of accurate learning. With both Dataset 1 (58 instances) and Dataset 2 (54 instances), six residues were consistently selected to produce the best learning performances (Table 1), using a KNN classifier and a transformed BLOSUM62 scoring matrix. We reason that the residues that were identified as most informative in learning are more likely involved in modulating the physical activation process of VKCs. The selected residues were mapped onto a schematic of the S1–S6 structure (Figure 4). All of them reside in S1–S3, a region that likely plays a modulating role in VKC functioning [39,40].

### Independent evolution of character states in informative characters

A phylogenetic tree was inferred with MrBayes v3.0b4 [41] using the 54 channel data set. The evolution of individual characters was then inferred on this tree using maximum parsimony criteria as implemented in MacClade [42]. In the case of all six of the informative characters, at least one of the character states has arisen multiple times during channel evolution (Table 3). In all cases, the residue identities that have arisen independently during channel evolution have large hydrophobic side chains (F, I, V, L).

## Discussion

### Learning with high dimensional data

Data with high dimensionality are a "curse" to learning performance. As a rule of thumb, the number of instances should be no less, and preferably more, than the number of features to obtain a reasonable learning accuracy [31]. Even with a large number of instances, a large number of irrelevant features can still compromise the learning performance [31]. For biological data, however, enough examples with relatively small dimension are not always achievable. Without feature selection or some other form of dimension reduction prior to data analysis, learning performance with high dimensional data is poor. Many dimension reduction methods have thus been applied to improve learning performance, including principle component analysis and linear discriminant analysis [43,44].

We faced this problem in our analyses. There were less than 60 VKC sequences with published V_50 _values, and there are nearly 300 residues in each sequence alignment after trimming poorly aligned regions. Most residues likely have little or no role in determining V_50 _values, and thus are "irrelevant features". Training without feature selection using several machine learning algorithms yielded categorical prediction accuracies consistently lower than 30% (Figure 2A).

Application of a filtering analysis before learning improved the accuracy marginally (Figure 2B). Filtering is a pre-learning data processing method based on evaluation of the information content of the dataset, and thus is independent of the training process. It has been successfully used in other tasks to obtain better learning performance [32,33]. However, it may or may not select truly relevant features, depending on the datasets and the selection criteria. Considering the number of features and number of instances in our datasets, some irrelevant features may well correlate with the final class labels by chance and display a high information gain potential that does not reflect a functional connection between sequence and V_50_.

We then applied a wrapper algorithm to select features during the learning process. Wrapper uses a heuristic search to select the subset or subsets of features that yield the best learning performance [31]. It "wraps" around the learner and selects best feature sets based on learning accuracies. In a heuristic search there is no formal guarantee that the search will not become trapped in a local optimum and miss the global optimum. To decrease the chance of missing the global optimum without making the analysis intractable, we selected the top 200 residue combinations at each round and used them all as starting points for the next round of searching. Best-first searching was continued until learning performance stabilized. The learning accuracies with wrapper increased greatly for all learning algorithms we used. The best categorical result was obtained with the KNN classifier (k = 1) with an accuracy of 60%. To generate a numerical predictor for V_50_, we also trained the KNN classifier combined with wrapper for numerical classification; the mean absolute error of prediction improved to 9.5 mV from 17.8 mV (Figure 2B).

Since wrapper does not use an exhaustive search and does not guarantee optimal feature selection, we applied "residue swapping" to explore the possibility that one or more residues would yield better results in the context of the finally selected residue set than they would in the initial search. However, residue swapping did not produce any feature sets that yielded significantly better predictive performance. Although, as an empirical operation, branch swapping does not guarantee the global optimum in phylogenetic analysis [45], this process is a reasonable heuristic approach for searching in the neighborhood of an optimum for other, better, feature sets.

### Outlier selection

Typically, with a sufficient amount of data, classification using machine learning is expected to be insensitive to outliers. However, a dataset with a low number of instances relative to features of structural and functional data increases susceptibility to outlier effects.

The V_50 _values in our dataset were obtained from publications from dozens of labs. We used averages for three V_50 _values in our datasets because different investigators have published different V_50 _values for the same VKC sequences. The difference in V_50 _values of same VKCs from different labs sometimes for two of those channels exceeds 15 mV [46-49]. Thus, it is almost certain that some VKCs in our datasets compromise learning because they are incorrectly labeled.

We evaluated the prediction accuracies with datasets from which one sequence was pruned at each round of training (Figure 3A). Based on the variations in learning performances, we stopped at Round 4 (Figure 3B). At both Round 2 and 4, learning performances displayed an improvement of MAE of almost 1.5 mV (Figure 3B). The improvement of learning performances after Round 4 decreased significantly (Figure 3B).

One sequence was deleted in each round of pruning. Creating the best learning performance from the remaining data, the top fifty such "remaining" sequence sets were all used as starting points for the next round of searching. Four sequences were consistently selected for "deletion" in the first four rounds, although in different orders. The best learning performance produced a MAE of 7.0 mV with the new dataset of 54 VKC sequences (Figure 2B), after deleting the four potential outliers; with the outliers the MAE was 9.5 mV.

Outliers may arise from experimental errors, or the channels may be activated by different mechanisms so V_50 _values would be affected by a set of residues that is different from those that affect the non-outliers. In the latter case, the "deleted" outliers become interesting research targets [50,51]. However, we could not rigorously exclude the possibility that they were selected as outliers due to the specific dataset we used and possible data over-fitting in our training.

Among the deleted outliers, VKC149, a squid Kv2 channel, was shown to undergo extensive RNA editing, leading to its functional diversity [52]. Its G-V curve, which was used to obtain its V_50 _value, had to be fitted with two Boltzmann functions, adding another layer of complexity to its gating mechanism [52]. VKC171 (Kv4.3 mouse) [50] is a fast inactivating channel. Its activation might overlap its inactivation, which would make it difficult to obtain an accurate V_50 _value [50].

We also tested the possibility channels with the most distant nearest neighbors might be outliers. We identified the nine sequences that were most distant from their nearest neighbors and sequentially removed them from the dataset, evaluating the MAE of the resulting predictor after each deletion using a 10 times 10-fold cross-validation. None of the four channels mentioned above was among this set. The MAEs of the first 8 deletion datasets were greater than that for the full set of 58 sequences (9.5 mV); after the ninth deletion the MAE was 9,2 mV. Thus, an *a priori *assumption that the most distant sequences will cause poor prediction performance is not valid in this case.

The predictor performance was also evaluated using the R-squared statistic and correlation coefficient of the predicted vs. the actual V_50 _values. Both of these measures showed a significant improvement of prediction performance with feature selection and outlier removal. The 10-times 10-fold cross validation of best predictor, using the six selected characters and Dataset 2, yielded an R-squared value of .77, indicating that the predictor was accounting for approximately 77% of the variation on V_50 _between channels. The linear correlation coefficient between the predicted and actual V_50 _values was 0.89, which also indicates that the predicted V_50 _values are well correlated with the actual values for the channels.

### Statistical evaluation using a permutation test

The machine learning approach that we have implemented searches for an optimal value of MAE, so it will always yield a model that associates the identity of some residues with the V_50 _value, even if that association is specious. To evaluate whether the association of residue identity and V_50 _is significant, a statistical test of significance is necessary.

A permutation test is a special case of randomization tests. With a small sample of data, a permutation test generates an approximate probability distribution for a null hypothesis. Permutation tests have been widely used in biomedical and other areas including microarray analysis, SNP research, and clinical studies [53-55]. Compared with other statistical analyses, a permutation test works well with small sample sets and it does not require a normal distribution, which many small samples do not have. Some researchers have even proposed that permutation test should be used in all cases [56].

We evaluated whether there is significant information linking the sequence of a VKC to its V_50 _value using one hundred permutations of the original dataset, where V_50 _values were randomly reassigned to sequences. One hundred different computational models were generated with one hundred different sets of features (residues). The best and worst MAEs among these permutation learning are 9.9 mV and 15.4 mV, respectively, with a mean MAE of 13.4 mV and standard deviation of 1.1 mV. The mean MAE, 13.4 mV, differs significantly from that of the predictor that was generated with the original, non-permuted, dataset, 7.0 mV (P < 10^-10^). Since both KNN classification and the feature selection process were involved in the permutation test, each test yielded a best model that mathematically correlates a set of residues with a permuted set of V_50 _values. The fact that the original model significantly outperforms any of the "permuted" models strongly supports the conclusion that the original learning has detected a valid association between sequence elements of VKCs and their V_50 _values.

### Evaluation of predictor using independent experimental data

Due to the limited number of data, we did not retain a portion of data as an independent test set when constructing the predictor. Instead, we used a repeated ten fold cross validation to estimate prediction errors on unseen data. To obtain an independent objective assessment of predictor performance, we located another thirteen VKCs that have been functional characterized, including VKCs that were recently cloned [36], as an independent test set. Using the predictor, the MAE of predictions of all thirteen new VKC instances is 9.7 mV (Additional file 2), which is higher than what we estimated using a repeated ten-fold cross validation (7.0 mV).

We also tested an alternative model, that the V_50 _could be predicted by assigning the average V_50 _value of the sister group of each channel on a phylogenetic tree that was constructed based on the full set of 296 aligned amino acid positions. We tested both a distance tree and a Bayesian maximum likelihood tree (Figure 6). The phylogeny-based predictions were significantly poorer than those we obtained with the fully optimized predictor (Additional file 2).

**Figure 5:**
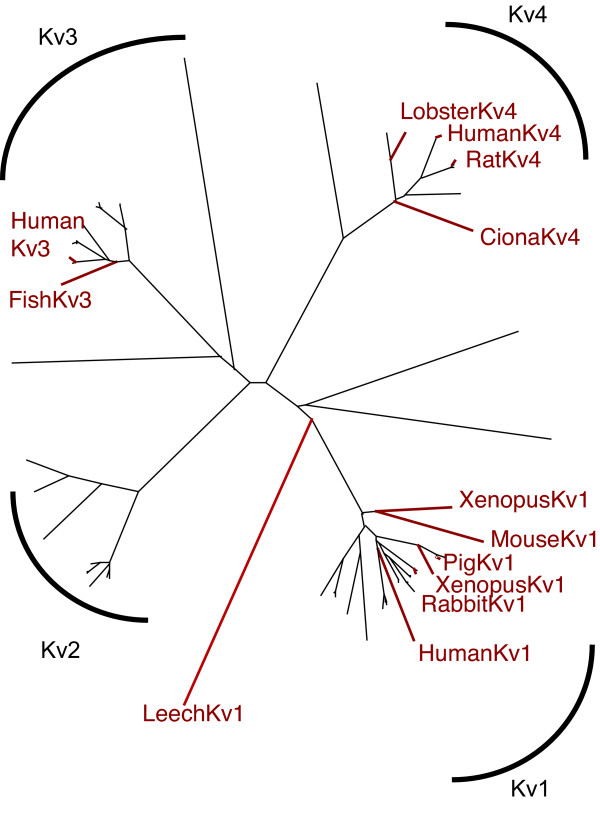
**Distance tree of training data and independent test data**. Distance tree illustrating the relative similarities of the 54 sets of six selected informative residues from the training set and the corresponding residues in the 13 sequences that were used for independent testing of the prediction performance. The names of the training set channels have been removed for clarity. Each of the four subfamilies form well supported clades, and are labeled as Kv1, Kv2, Kv3, and Kv4. The branches and names of the wild type VKC channels from the independent test set are in red. Most test VKC sequences are clustered closely with one or more channels from the training data. Two VKCs from Hirudo medicinalis (LeechKv1) and Ciona intestinalis (CionaKv4), have long branch lengths.

**Figure 6:**
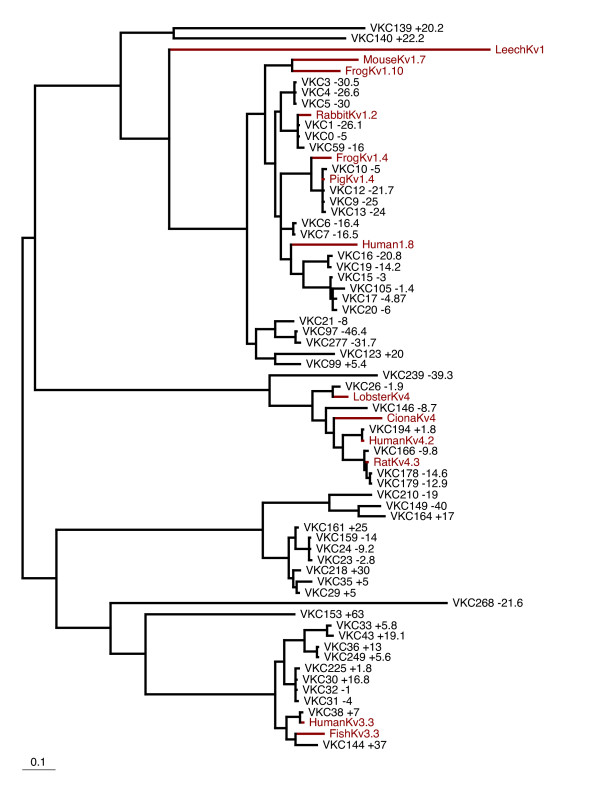
**Bayesian maximum likelihood tree of training data and independent test data**. The set of 54 VKC sequences used for the final predictor and the 13 VKC sequences from the independent test set were aligned using MUSCLE [70] and the alignment was trimmed to 296 residues. This data set was used to generate a maximum likelihood tree using MrBayes [41], using a total of 500,000 cycles with a 250 cycle burn-in. The training set channels are labelled with their VKCDB identification number and their V_50 _value, the 13 test sequences are labelled with the name of the species and the channel, and were highlighted in red (Figure 6).

The optimal predictor was built with a KNN classifier. In KNN classification, a close "neighbor" from the training set will be used as a template to classify a new instance. If the training data are not evenly distributed in the instance space, some areas contain fewer instances with larger empty space than others, as is shown in the distance tree of the training data (Figure 5). Evidently, instances that are in these sparse areas will likely be less accurately classified, since they do not have close neighbors. In fact, superposition of the test VKC data on the distance tree of the training data clearly showed an unequal distribution in the sequence space (Figure 5). Among the thirteen test VKC data, all but two are from species that exist in the training set. One VKC is from *Hirudo medicinalis *[37] and the other is from *Ciona intestinalis *[36]. The difference between the *H. medicinalis *channel sequence and its nearest neighbor in the training set is much greater than for any other sequence and its nearest neighbor. The prediction errors of these two VKCs using the predictor are 27.3 mV and 27.4 mV, respectively. When the *H. medicinalis *sequence is removed from the analysis the MAE for the test set is 8.3 mV, and if both of these sequences are removed the MAE is 6.6 mV. This analysis indicates that a more phylogenetically diverse selection of channels in the training set should improve prediction performance.

In the training set, all V_50 _values were determined from channels expressed in *Xenopus *oocytes. In the test set, however, we also included VKCs that have V_50 _values determined in other cells, such as HEK293 and CHO cells [35,57]. Although it is known that the experimental V_50 _values of VKCs can vary if they are measured in different cells, the difference is often not significant, as shown by experimental data of several VKCs that have been characterized in both *Xenopus *oocytes and other cells. Therefore, we believe that the test set serves as a valid independent test set. In fact, it is likely that a better estimate would be obtained if all test instances were measured in *Xenopus *oocytes, which would remove variation due to differences in expression systems.

We also compared experimental data from a mutagenesis scanning study by Li-Smerin *et al *with predictions by our predictor (Table 2) [38]. Despite using a test set comprising results from VKC mutants, and the presumably drastic difference between data distributions of the test set and our training sets, prediction results are consistent with results from the published mutagenesis study of VKCs (Table 2). This result supports the conclusion that our estimated prediction error is close to the true error.

Although all of the mutations have changed one of the six informative residues, only two of them have mutated to an amino acid that is represented at that position in one of the training set sequences. The V_50 _predictions for these two mutants, marked with asterisks, are much closer to the observed V_50 _values than those for the other three mutants. This suggests that as more channels are cloned and characterized and the variety of the training set increases the performance of this kind of machine learning and prediction will improve.

Little variation of voltage sensitivity from the wild type (Kv2.1 rat) was predicted for most VKC mutants (Table 2). These mutants were shown to have little impact on voltage sensitivity if they were mutated to Ala in Kv2.1 [38]. Consistently, these mutants were predicted to have a V_50 _value close to that of the wild type (Table 2). One mutant, A154Y of Kv2.1 rat, displayed a large shift of V_50 _of over 10 mV [38], and our predictor also predicted a large positive shift in V_50 _value (Table 2). Although this is the largest margin between the predicted V_50 _and the experimental data, the correct prediction of direction in V_50 _shift by our predictor is encouraging.

### Identification of biologically important residues (features)

One goal of building a model that can predict V_50 _value of a given VKC sequence with reasonable accuracy is to identify residues that are involved in modulating voltage sensitivity. In our analysis, different feature (residue) sets were selected by wrapper and screened to identify the feature set that yielded the best learning performance. In a forward selection approach, one feature (residue) was added at each round. Although different features were sequentially selected in different orders during the first five rounds, the feature set that produced the best learning performance converged to six residues. These six residues are the best features in predicting the V_50 _value of VKCs based on their amino acid sequences. The prediction was also validated by independent experimental data (Additional file 2 and 2). Likely, these residues are central to setting the voltage sensitivity of VKCs.

Some functionally important residues may not be identified using our approach. If a group of residues co-vary because they interact with each other to affect V_50 _values of VKCs, for example, after one residue is identified, the addition of the other residues may not further improve learning performances, and thus they would not be selected. However, no feature co-varied precisely with the six selected features in a covariation analysis (not shown). Also, our datasets contain a tiny subset of all the VKCs in nature that may not be an unbiased representation of all VKCs, so the residues that are selected may be only pertinent to these specific datasets. The quality of the experimental data are also a factor, indicated by the different V_50 _values obtained by different research labs for a same VKC [46-49]. Outlier selection may have helped alleviate the problem, but it is still a potential error source. Nevertheless, the combination of the selected residues should be a good indication of potentially functionally important structure elements.

The positions of the six features selected with a modified BLOSUM62 matrix are shown mapped onto a schematic of the VKC channel in the membrane in Figure 4A. All six selected residues are on one of the transmembrane helices S1, S2 or S3, none are found on the primary voltage sensor, S4, or on the pore/gate complex S5–S6. Although the S1–S3 region is not the primary voltage sensor, there is extensive experimental evidence that interactions between the S4 helix and the S1–S3 region are important in setting the value of V_50_. Tiwari-Woodruff et al. [58,59] and Papazian et al. [60] have demonstrated that three acidic residues in S2 and S3 (E283, E293 and D316) interact with basic residues in S4 during the process of voltage response, and that altering these residues in the *D. melanogaster *Shak channel will alter the value of V_50 _by more than 40 mV [61]. Those results suggest that one face of both S2 and S3 face the charged surface of S4 at least during the process of activation, and possibly constitutively. There is also a highly conserved acidic residue at the C-terminal limit of S1. Of the six residues that have been identified as relevant features in the current study, four are exactly two residues before or after one of these highly conserved acidic residues in the sequence. As illustrated in Figure 4B, this spacing along an alpha helix places the variable residues that we have identified approximately on the opposite face of the helix from the charged residues that have a demonstrable role in setting V_50_.

The variation of the six selected residues are mostly limited to nonpolar, hydrophobic residues including Ile, Leu, Val, Phe, and Ala, although there are several incidences of polar residues and one charged residue, the His at position 117 of the data in all Kv3 (*Shaw*) channels)[62] (Table 3). Thus, it appears that the observed variations in these six residues are responsible for many of the small energetic differences between channels that are responsible for differences in V_50 _values; whether these energy differences involve intramolecular interactions between several helices of a single channel protein or interactions between the helices of the protein and the surrounding hydrophobic layer of the lipid bilayer is unknown.

Many residues of VKCs that are responsible for voltage sensing and selective ion permeation are charged or polar amino acids, which generate relatively strong ionic interactions [40]. Variation of residues involved in strong ionic and bonding interactions often lead to, if not inactive channels, drastic variation in function [40]. Hydrophobic interactions among nonpolar residues or between nonpolar residues and lipids, on the other hand, are often energetically of lower magnitude and thus variations in these interactions will cause quantitatively small variations in functional parameters. These hydrophobic interactions can yield a practically continuous range of interaction energy magnitudes. Small variations in these kinds of residues are less likely to cause functional disruption, but are more likely to play "secondary" roles in VKC functioning and help tuning and shaping the sensitivity of different functional properties. The nonpolar hydrophobic features of the predicted voltage modulating residues are consistent with a role in modulating the targeted functional feature, the voltage sensitivity of VKCs.

The extant models for voltage sensing all focus on the movement of the voltage sensing S4 transmembrane helix and how it may alter the conformation of the pore and the S5 and S6 helices to open and close the ion channel. In most cases, the consequent movements of other parts of the molecule are not explicitly addressed. The "paddle" model based on the structure of the KvAp channel [11,63] predicts that the C-terminal half of the S3 helix moves in concert with the S4 helix during gating. However, both of the informative residues in the S3 helix are in the N-terminal half of S3 and so would not be directly involved in the primary gating movement.

The results of the current study indicate that as the interactions of S4 with the other transmembrane helices change, the interactions of those helices with each other or the lipid bilayer, or both, are also changing. These changes in interaction are in turn responsible for the small incremental differences in the V_50 _value for the channel. This analysis neither confirms nor rejects any of the current models of voltage response. It does predict that that when these models are formulated more precisely, the energy differences between the open and closed states must take into account hydrophobic interaction energies.

One possible confounding effect in a comparative study is the possibility of evolutionary hitchhiking, the possibility that the residue identities are correlated with functional variation by virtue of having been independently fixed in an ancestral population at the same point as a voltage variation evolved. However, if that were the case, then it would be expected that evolutionary reconstruction of the individual features would not show multiple independent origins of specific residues. This is not the case for the features selected in this analysis. Each of the six selected residue positions has evolved independently to specific residue identities (Table 3).

In a recent study pairs of residues that co-vary during evolution and are presumably involved in the essential functions of VKCs, were computationally identified. Most of these residues are located in the so-called core functional elements (S4–S6) [2], the pore region and the voltage sensor. Our approach to structure/function analysis is aimed at identifying structural elements that modulate the voltage sensitivity, not those that are essential for voltage sensitivity. While S4 is considered the main voltage-sensing unit, S1–S3 is thought to play a modulating role in the voltage sensitivity of VKCs [39,40]. Consistent with their modulating roles, all residues selected in our study are indeed located in S1–S3 region (Figure 3). It is not surprising that other residues in the S1–S3 region that were not selected in the present study have been shown to modulate the voltage sensitivity of VKCs [59,60]. Most of these highly conserved residues appear to interact directly with the positively charged key residues in the voltage sensor (S4 helix) through charge interactions. These residues are highly conserved among VKCs and so do not co-vary significantly with V_50 _values in most channels.

## Conclusion

Machine learning methods have been widely used in biological analyses because of their capacity for dealing with data-rich tasks. Using a dataset of 58 VKC sequences with their V_50 _values, we built a predictor that predicts the V_50 _value of a given VKC based on its amino acid sequence. Despite the limited number of training data, and the uncertain quality, an MAE of prediction of 7.0 mV was obtained using a KNN classifier combined with a wrapper for feature selection (Figure 2D). The prediction accuracy was evaluated by a repeated (ten times) ten-fold cross validation. It is also validated by V_50 _prediction from independent experimental data (Table 2 and Additional file 2). As more data become available from ongoing isolation and characterization of novel VKCs, better prediction is expected. During training, four possible outliers were singled out and removed from the training set to improve the learning performance (Figure 3). Several residues with potential biological implications were identified for further study (Figure 4).

The analysis presented in this report demonstrates how machine learning methods can be productively applied to structure-functional study with datasets of limited size. These analyses can predict certain biological functions with a reasonable accuracy and can identify potentially functionally important residues for experimental testing of specific hypotheses of the structure/function relationship in a family of proteins.

## Methods

### Dataset

Data used in this project were drawn from VKCDB, a voltage-gated potassium channel database [34]. Although VKCDB has over 350 channel sequence entries, only 58 VKC sequences have associated half activation voltage (V_50_) values. Sequence and V_50 _values for these channels were extracted from VKCDB; most of the sequences have more than 500 amino acid residues. The published V_50 _values have been experimentally determined under similar experimental conditions, using a two-electrode voltage clamp in *Xenopus *oocytes [24]. Averages were used for those VKCs for which different V_50 _values have been published by different groups [46-49].

All sequences were aligned with PepTool [64], followed by manual adjustment. Because there is large sequence variation at both termini and some loop regions of the VKCs, only blocks of residues that contained relatively few gaps were kept for analysis (Dataset 1, see Additional file 1).

### Independent test dataset

Thirteen wild type VKCs with experimentally determined V_50 _values were used to obtain an objective assessment of the predictor (see Additional File 1). The V_50 _values of these VKCs had been determined in several different cell hosts including *Xenopus *oocytes, HEK293 cells, and CHO cells [34-36]. Another six VKC mutants with V_50 _values determined in an Ala-scanning mutagenesis experiment [38] were also used to evaluate the predictor.

### Problem formulation

To formulate our problem into a typical supervised learning task, the dataset was considered as a training set with 58 instances. Each of the alignment positions was taken as one nominal attribute (feature), and all attributes were assumed to be independent of each other. In numerical prediction analyses, the classes were the real V_50 _numerical values. In categorical prediction analyses, V_50 _values were divided into seven nominal classes based on their values; -50 > V_50 _≥ -30 mV, -30 > V_50 _≥ -20 mV, -20 > V_50 _≥ -10 mV, -10 > V_50 _≥ 0 mV, 0 > V_50 _≥ 10 mV, 10 > V_50 _≥ 20 mV and 20 > V_50 _≥ 65 mV. The goal is to extract the data model that can best describe the relationship between the (attributes) features and the labeled classes of these data, and correctly predict the class or the numerical value of V_50 _of any given VKC sequence.

### Basic learning algorithms

The KNN (k-nearest neighbor) classifier was used in both numerical prediction and categorical prediction analysis. All KNN classifications were tested with k values of 1 to 5. Decision Tree, Naïve Bayes Learner, Kernel Density Classifier and OneR Classifier algorithms were also used in categorical predictions. The algorithms used are implemented in the WEKA package 3.2.3 [30].

The prediction accuracies were used to evaluate the learning performance in categorical prediction. The mean absolute errors (MAEs), the average absolute difference between the predicted values and the published values, were used to assess the numerical prediction. All learning performances were evaluated using a repeated ten times ten-fold cross validation.

### Feature selection

Filtering and wrapper algorithms were used to select a subset of features with the best prediction performance, to decrease the dimensionality of the learning problem.

For filtering, features were ranked by information gain [32,33,65], then different numbers of top-ranked features were selected for learning, and the sets that produced the best learning performance were considered the best feature sets using filter.

The wrapper approach to feature selection screens subsets of features in a dataset and selects the "relevant" features based on learning performances [31]. Forward selection was used in this approach. In the first round of analysis each individual residue was evaluated for its prediction quality (lowest MAE in 10 times 10-fold cross validation) and the 200 residues with best predictive performance were retained. In subsequent rounds additional features (residues) were added at each round, the prediction quality of the resulting subsets of residues were evaluated, and the 200 subsets with best prediction quality were retained for the next round of feature addition. This process was repeated until learning performances stopped improving [31]. Despite the existence of redundant feature sets at each round, the number of non-redundant feature sets was well above 100 at each round. The search was continued for five rounds after the learning performance stopped improving to ensure that performance had reached a plateau.

### Residue swapping

We also applied a "residue swap" heuristic, similar to the branch-swapping step during the construction of phylogenetic trees [45], to try to further improve the prediction accuracy. For the best feature set selected by the wrapper algorithm, each residue was sequentially replaced with every other residue that was not in the final set, and the new feature combination was evaluated for prediction accuracy using a repeated ten-fold cross validation.

### Distance matrices in k-nearest neighbor classification (KNN)

A KNN classifier is a set of n-dimensional vectors (where n = the number of features) to which new instances are compared [25]. It classifies a new instance by evaluating its distance from each of the classifier instances and chooses the class label of the classifier instance that is closest to the new instance as the predicted class of the new instance. For more than one classifier instance with an identical distance to the new instance, one of the class labels of these classifier instances is randomly picked and assigned in categorical predictions; averages of equidistant classifier instances are calculated for numerical prediction.

The Euclidean distance between any two vectors is obtained by taking the sum of the square of the distances between all pairs of attributes (dimensions), on the assumption that the sites are independent and therefore their dimensions are orthogonal. For nominal attributes, such as amino acid residues, the KNN algorithm can simply takes 1 and 0 as the distance between a pair of different and same residues, respectively. We also implemented the KNN algorithm to incorporate PAM [66] and BLOSUM [67] matrices as a measure of distance between pairs of features (residues) of two VKC sequences (Formulas 1.1 and 1.2). Since the scores in amino acid comparison matrices go up when two amino acid residues are more similar to each other, which is the opposite of distance measurement in KNN classification, we converted amino acid comparison scores accordingly (Formula 1.0). In all cases any gap was scored as the maximum distance for the relevant scoring matrix.

In BLOSUM62 or PAM100 matrix:

converted score_i _= matrix_maximum_value - original_score_i _    (1.0)



D: Distance between two instances.

n: Number of features.



Other matrices: score_*i *_= converted score_i _from pairwise comparison     (1.2)

### Outlier selection

To minimize the effect of possible outliers, a best-first search was performed. One VKC sequence was deleted from the training set at each round, and the learning was carried out with the remaining VKC sequences. The deleted sequence was considered an outlier if the remaining dataset yielded better learning performance than the full dataset. The search stopped if the learning performance no longer improved after a further round of deletion. Due to computational complexity, the outlier selection was not combined with full feature selection of wrapper [31]. Instead, the best feature set selected by the wrapper algorithm was applied to outlier selection.

### Permutation test

We randomly shuffled the classes of each instance in Data Set 2 (the 54 sequences remaining after outlier selection) to produce 100 permuted datasets. With each of these datasets, training was repeated using KNN classification combined with wrapper, with identical parameters and settings as in the original training. This process was repeated one hundred times and the MAE of each of the predictors was collected.

### Final predictor construction

After removal of four outliers from the original dataset, the remaining 54 sequences formed a new dataset (Dataset 2) that was used to develop the final predictor. During the training process using Dataset 2, one best feature (residue) set was selected by wrapper to predict the V_50 _values with an MAE of 7.0 mV. One predictor was then constructed, using Dataset 2, the best feature set, the BLOSUM 62 scoring matrix and the KNN classification (k = 1).

To predict the V_50 _value of a new query sequence, the query sequence is first aligned with the profile alignment of Dataset 2 using ClustalW [68]. The residues at the aligned selected positions are extracted to produce a data file for V_50 _prediction.

### Phylogenetic reconstruction and distance tree construction

The training data were used to construct a phylogenetic tree using MrBayes v. 3 [41]. Reconstruction of the evolution of the character states in the six selected features was then evaluated on this tree using maximum parsimony as implemented in MacClade [42].

The relative distances of the 54 VKCs in the training set (Dataset 2) and the 13 independently characterized test sequences were evaluated by summing the distances for each of the six selected features. A distance tree was then constructed using PAUP*4 [69] (Figure 5). The names of the 54 sequences in the training set are not shown, for clarity. The branches connecting the 13 test sequences to the tree formed by the training set are highlighted in red, and the names of those 13 sequences are shown.

## Authors' contributions

BL did the data collection and performed the computational analyses. WJG initiated the project, supervised the execution of the analyses and provided regular evaluation of the outcomes. Both of authors shared in the preparation of the manuscript.

## Supplementary Material

Additional File 1 TrainingandTest.nexus is a plain text file in NEXUS format, containing the sequence data matrix that was used for the machine learning analysis and the 13 channel sequences that were used for validation of the predictor performance. Click here for file

Additional File 2 Table 2. Click here for file

## Figures and Tables

**Table 1:** "Best" feature (residue) sets selected by wrapper with different distance matrices.

Distance matrices	Selected residue sets
BLOSUM62	97, **100**, 117, 125, 135, **154**
PAM100	83, 95, 97, **100**, 117, 131, 141, **154**
Identity matrix	83, 92, 95, **100**, 103, 123, 135, **154**, 273

Wrapper feature selection identified different, but overlapping, sets of informative residues, depending on the distance matrix that was used. Residues that were selected with more than one matrix are underlid, and residue **100 **and **154 **(in bold) were selected with all three distance matrices.

**Table 2:** Published mutant data and predicted V_50 _values.

**Ala scan (Kv2.1)**	Published V_50 _(mV)	Predicted V_50 _(mV)	Wild type (mV)
**L97A**	0.6	-7.23	-4.9
*I100A	-7.3	-7.23	-4.9
**L117A**	-1.6	-7.23	-4.9
*V125A	-4.6	-7.23	-4.9
**L135A**	1.5	-7.23	-4.9
**A154Y**	7	27.5	-4.9
**MAE**	**7.5**		

The V_50 _predictions for a set of published alanine scanning mutants are compared to the published measured V_50 _values. The residues marked with * are residues in which an alanine exists at this position in at least one of the channels in the training dataset.

**Table 3:** Amino acid residues at the six positions selected as most informative in our dataset.

Position	Residue (number of independent origins)
97	*C (1), F (2), I (4), L (A), V (5), *Y (1)
100	A (1), *F (1), *G (1), I (A), *L (1), *T (1), V (4)
117	H (1), I (4), L (A), V (1)
125	A (A), *C (1), *F (1), I (3), L (2), T (1), V (3)
135	I (4), L (A), *T (1), V (2)
154	A (1), *C (1), *F (1), I (A), L (2), *M (1), V (5)

The amino acid residues that are present at each of the six informative positions of the 56 channels used for construction of the final predictor are presented. Residues marked with an asterisk only occurred once at that position in the training data set. The number in brackets represents the number of independent origins of that residue, that is the minimum number of times that the residue evolved independently at that position. An A in brackets indicates that this is the inferred ancestral residue identity.
