# Supplementary material for: Computational identification of residues that modulate voltage sensitivity of voltage-gated potassium channels
Source: BMC Struct Biol. 2005 Aug 19;5:16. doi: 10.1186/1472-6807-5-16 (PMC1208917; doi:10.1186/1472-6807-5-16)
Supplement: Additional File 2 — Table 2. [file 1472-6807-5-16-S2.pdf]

<table>
<tr><th>Channels</th><th>Published V_{50} (mV)</th><th>Predicted V_{50} (mV)</th><th>Nearest neighbors</th><th>Phylogenetic Prediction</th></tr>
<tr><td>Kv1.2 rabbit</td><td>-19.6</td><td>-16.45</td><td rowspan="2">Kv1.2 human –5
Kv1.2 mouse –26.1
Kv1.6 rat –14.2
Kv1.6 human –20.8
Kv1.2 dog -16</td><td>-15.7</td></tr>
<tr><td>Kv1.7 mouse</td><td>-8</td><td>-16.45</td><td>-16.3</td></tr>
<tr><td>Kv1.4 pig</td><td>-17</td><td>-18.92</td><td rowspan="2">Kv1.4 ferret –25
Kv1.4 human –5
Kv1.4 rat –21.7
Kv1.4 mouse -24</td><td>-18.9</td></tr>
<tr><td>Kv1.4 frog</td><td>-26</td><td>-18.92</td><td>-18.9</td></tr>
<tr><td>Kv1.8 human</td><td>3.6</td><td>-3.8</td><td>Kv1.5 rabbit –4.87
Kv1.5 rat –3
Kv1.5 human –6
Kv1.5 bovine –1.4</td><td>-8.4</td></tr>
<tr><td>Kv1.10 frog</td><td>-11.3</td><td>-8</td><td>Shaker fly –8</td><td>-16.3</td></tr>
<tr><td>Kv3.3 human</td><td>11</td><td>6.5</td><td rowspan="2">Kv3.1 rat 16.8
Kv3.1 human –4
Kv3.1 mouse –1
Kv3.4 rat 5.8
Kv3.4 human 19.1
Kv3.3 rat 7
Kv3.1 dog 1.8</td><td>7.0</td></tr>
<tr><td>Kv3.3 fish</td><td>15.6</td><td>6.5</td><td>37.0</td></tr>
<tr><td>Kv4 lobster</td><td>-19</td><td>-7.4</td><td rowspan="3">Shal fly –1.9
Kv4.3 ferret –9.8
Kv4.3 human long –14.6
Kv4.3 human short –12.9
Kv4.3 rat 1.8</td><td>-1.9</td></tr>
<tr><td>Kv4.2 human</td><td>-3.2</td><td>-7.4</td><td>1.8</td></tr>
<tr><td>Kv4.3 rat</td><td>5</td><td>-7.4</td><td>-13.8</td></tr>
<tr><td>MAE</td><td colspan="3">6.6</td><td>9.4</td></tr>
<tr><td>*Kv1 leech</td><td>8.3</td><td>-19</td><td>Shab fly -19</td><td>-17.6</td></tr>
<tr><td>*Kv4 Ciona</td><td>20</td><td>-7.4</td><td>Shal fly –1.9
Kv4.3 ferret –9.8
Kv4.3 human long –14.6
Kv4.3 human short –12.9
Kv4.3 rat 1.8</td><td>-9.0</td></tr>
<tr><td>MAE</td><td colspan="3">9.7</td><td>12.2</td></tr>
</table>
